# Temporal Changes in Inflammatory Markers and Laboratory Parameters in Relation to Risk of Neonatal Sepsis

**DOI:** 10.3390/jcm15197625

**Published:** 2026-10-01

**Authors:** Karolina Tądel, Andrzej Dudek, Agnieszka Drozdowska-Szymczak, Paweł Krajewski, Magdalena Rutkowska, Iwona Bil-Lula

**Affiliations:** 1 Department of Medical Laboratory Diagnostics, Faculty of Pharmacy, Wroclaw Medical University, 211 Borowska Street, 50-556 Wroclaw, Poland; 2 Department of Econometrics and Informatics, Faculty of Economics and Finance, Wroclaw University of Economics, Nowowiejska Street, 58-500 Jelenia Góra, Poland; 3 Department of Neonatology and Neonatal Intensive Care, Institute of Mother and Child, Kasprzaka 17a, 01-211 Warsaw, Poland; 4 Department of Epidemiology and Biostatistics, Institute of Mother and Child, Kasprzaka 17A, 01-211 Warsaw, Poland

**Keywords:** neonatal sepsis, temporal changes, biomarkers, disease progression, inflammatory markers

## Abstract

**Background/Objective:** Early diagnosis of neonatal sepsis remains a challenge due to the nonspecific nature of clinical symptoms and the delayed dynamics of conventional inflammatory markers. The traditional diagnostic approach is based on static cut-off points, which often do not consider the nonlinear nature of the inflammatory response. The aim of this study was to identify dynamic variables associated with sepsis progression, constituting the foundation for future artificial intelligence (AI) models. **Methods:** A retrospective analysis was performed using anonymised clinical and laboratory data from a cohort of newborns (*n* = 122) with suspected sepsis, along with maternal clinical data. Using feature engineering, descriptive statistics parameters were generated for selected parameters, including rates of change in the 24 h and 48 h time windows. This was combined with the verification of clinical and perinatal parameters that affect the assessment of clinical status and diagnosis of sepsis. The correlation analysis was performed using Spearman’s rank correlation coefficient (*p* < 0.05). **Results:** Dynamic parameters showed significant associations with sepsis progression than static measurements. The highest correlation was recorded for the rate of CRP growth (CRP_abs_change_rate_max, r = 0.613, *p* < 0.001), compared with the maximum values (CRP_value_max, r = 0.591, *p* < 0.001). High correlations were also observed for leukocyte parameters, especially neutrophils (NEUT_%_pct_change_rate_max, r = 0.547, *p* < 0.001) and basophils (BASO_%_value_max, r = 0.606, *p* < 0.001), with the strongest correlations observed within the first 24 h (r > 0.70). **Conclusions:** Neonatal sepsis progression may be more comprehensively characterised by temporal changes in laboratory parameters, not by their absolute values. Rates of change in inflammatory markers may provide clinically relevant complementary information on the course of the disease, reflecting the complex and nonlinear nature of the inflammatory response. The results indicate that the analysis of the trajectory of changes in parameters provides a promising basis for the future development and validation of predictive models based on artificial intelligence for earlier and more accurate diagnosis of neonatal sepsis.

## 1. Introduction

### 1.1. Epidemiology of Sepsis in Neonatology

Sepsis remains one of the most serious challenges in modern neonatology and is a leading cause of neonatal morbidity and mortality worldwide. According to the Global Burden of Disease Study, neonatal infections account for approximately 15% of all deaths among children under 5 years of age, representing the predominant cause within this group [1]. It is estimated that approximately 3 million cases of neonatal sepsis occur worldwide each year, of which up to 500,000–700,000 are fatal, primarily in low- and middle-income countries [2].

In highly developed countries, the prevalence of early neonatal sepsis (early-onset sepsis, EOS) is 0.5–1.0 per 1000 live births among full-term newborns, rising to 10–15 per 1000 among premature infants with a birth weight below1500 g [3]. EOS is characterised by the onset of symptoms within the first 72 h of life. Late-onset sepsis (LOS), which occurs after 72 h of life, occurs in 10–30% of newborns with very low birth weight hospitalised in neonatal intensive care units (NICUs) [4]. The mortality rate associated with neonatal sepsis in developed countries is 5–10% for LOS, however, mortality may exceed 30–40% in cases caused by Gram-negative bacteria or complicated by septic shock [5].

In addition to being directly life-threatening, neonatal sepsis is associated with severe health complications and long-term consequences. Cohort studies have shown that children who have experienced sepsis in the neonatal period have a 2–3 times higher risk of neurodevelopmental disorders, including cerebral palsy, cognitive delay and hearing loss [6]. Neonatal sepsis also imposes a substantial economic burden on healthcare systems and is estimated to range from $10 billion to $469 billion per year, considering both the direct costs of healthcare and the long-term impact on society [7].

### 1.2. Laboratory Diagnostics and Microbiological Gold Diagnostic Standard

The diagnosis of neonatal sepsis remains a major clinical challenge due to the nonspecific nature of its symptoms and the limitations of currently available diagnostic methods. Symptoms of sepsis in newborns, such as thermal instability, tachycardia, respiratory disorders, acid–base metabolism disorders or food intolerance, overlap with those observed in many noninfectious conditions, including respiratory disorders, necrotising enterocolitis or metabolic disorders [8].

Traditional diagnostics is based on the so-called cut-off values for markers such as C-reactive protein (CRP), procalcitonin (PCT) or interleukin-6 (IL-6). C-reactive protein (CRP), which is an acute phase reactant, is synthesised in the liver under the influence of IL-6. Procalcitonin (PCT) demonstrates more rapid kinetics, with serum levels increasing within 6–12 h, but its interpretation in newborns is hampered by a physiological increase in concentration in the first 48 h of life [9]. CRP is the most widely used marker in clinical practice. Interleukin-6 (IL-6) is considered the earliest detectable biomarker, with levels rising within 2–6 h after infection onset, but its short half-life (approx. 2–4 h) substantially limits its diagnostic utility [10]. However, single measurements taken at irregular hourly intervals are characterised by a low positive predictive value (PPV) in the first hours of infection [11]. The delayed rise in CRP levels, ranging from 12 to 24 h from the inflammatory stimulus, creates a dangerous diagnostic window in which decisions about the patient’s treatment, including antibiotic therapy, are often made subjectively [12,13].

Although laboratory biomarkers remain an important component of neonatal sepsis diagnostics, current neonatal practice increasingly emphasises clinical assessment and continuous non-invasive monitoring as the basis for early recognition of patient deterioration. Due to the vulnerability of neonatal patients and the need to minimise unnecessary blood sampling, laboratory testing should be performed selectively and in a clinically justified manner. Therefore, dynamic evaluation of clinical symptoms and routinely monitored physiological parameters remains central to contemporary sepsis assessment. Moreover, maternal and perinatal data may provide additional diagnostic value and should be integrated into future predictive models. However, despite the importance of clinical assessment and supporting perinatal information, microbiological confirmation remains a key component of the diagnostic process. The waiting time for the culture result is typically 24 h to 72 h with a full antibiogram, which delays definitive diagnosis and may affect therapeutic decisions [14]. This phenomenon has contributed to the emergence of the concept of clinical sepsis, which may account for up to 50–60% of all diagnosed cases in this population [15]. The limited sensitivity of blood cultures is due, among other factors, to low blood sample volumes, prior initiation of antibiotic therapy and low levels of bacteremia. Insufficient blood sample volume may contribute to false-negative culture results. Studies have shown that approximately 1 mL of blood is needed to achieve adequate pathogen recovery in neonatal blood culture diagnostics [16]. These limitations highlight the need for additional diagnostic tools, including analyses of biomarker relationships and data-driven methods.

### 1.3. Limitations of Conventional Diagnostic Approaches

A fundamental limitation of the traditional diagnostic approach is its point nature, which is based on single marker measurements interpreted in relation to constant reference values. This approach does not take into account the dynamic nature of the inflammatory response, in which the direction and rate of biomarker changes over time may be more clinically relevant than absolute values measured at a single time point [17]. The concept of analysing the kinetics and trajectory of marker changes has already been applied in other fields of medicine. In cardiology, the dynamics of troponin concentration is a diagnostic criterion for acute myocardial infarction [18]. In oncology, the PSA doubling time is a recognised prognostic factor in prostate cancer [19].

In the neonatal population, data regarding dynamic biomarker analysis remain limited. Procalcitonin serial determination has been shown to have a better correlation with response to treatment than CRP and leukocyte count in monitoring neonatal sepsis therapy [20,21,22]. The recently introduced marker presepsin (sCD14-ST) exhibits promising kinetic properties and studies have shown that combining parameters, including presepsin or IL-6 and CRP, achieved an AUC of 0.97 for the diagnosis of EOS [23,24].

Modern neonatology is increasingly shifting from static to dynamic data analysis. The use of markers with faster kinetics, such as interleukin-6 (IL-6), in serial measurement models allows for earlier detection of the inflammatory response and a better reflection of its dynamics over time [25]. IL-6 is one of the markers of the early phase of the inflammatory response, the concentration of which increases during the first hours of infection, ahead of classic markers of the acute phase, such as CRP [26].

Nevertheless, most studies to date have evaluated biomarkers using conventional approaches by comparing point values between groups or using single cut-off values. There is a lack of systematic studies evaluating high-resolution temporal biomarker trajectories and defining optimal input variables for predictive models.

### 1.4. A New Direction, Predictive Medicine and Artificial Intelligence (AI)

In the era of digitisation of medicine, serial laboratory data is an ideal contribution to machine learning (ML) models. The development of information technology and artificial intelligence (AI) methods opens new possibilities in the diagnosis of neonatal sepsis. Machine learning algorithms demonstrated the ability to integrate multidimensional clinical and laboratory data to predict sepsis with an accuracy that exceeds traditional statistical models [27,28]. However, the effectiveness of ML models is directly dependent on the quality and characteristics of the input variables of the “garbage in, garbage out” principle [29]. The integration of multidimensional temporal laboratory data with monitoring of vital signs (HRV, saturation) opens the way to the creation of AI-based early warning systems [30]. This work lays the foundation for the future development of AI algorithms by defining the key conclusions and overall perspective from the analysis of input variables needed to model sepsis progression.

### 1.5. Objectives

The main objective of this study is to determine critical input variables based on laboratory marker dynamics for assessing the clinical evolution of neonatal sepsis progression. The specific objectives follow:

- Characterisation of temporal trajectories of biochemical and haematological biomarkers, and selected clinical and perinatal parameters important for the assessment of sepsis progression in newborns with confirmed sepsis compared to newborns with and without suspected infection;
- Identification of optimal time intervals for serial monitoring of selected markers;
- Recognition of combinations of static and dynamic variables showing the strongest associations with the clinical course of neonatal sepsis in the first hours of life.

The influence of selected clinical and perinatal factors, such as exposure to antibiotic therapy before and on the day of birth, duration of membrane rupture group B streptococcal colonisation (GBS), birth weight, mode of delivery, prenatal steroid therapy and early signs of instability, including tachycardia and metabolic acidosis, was taken into account. The integration of these variables allows for the creation of a multidimensional feature space that combines biochemical and clinical data, which increases the predictive potential of the analysed model. It was hypothesised that dynamic biomarker-derived features, in combination with selected clinical and perinatal parameters, would provide clinically relevant complementary information beyond individual static measurements.

## 2. Materials and Methods

### 2.1. Study Design

The study was designed as a retrospective cohort study, aimed at assessing the dynamics of changes in inflammatory parameters and selected clinical and perinatal variables in newborns with suspected or confirmed neonatal sepsis. No dedicated quantitative sepsis progression score was used. Serial biomarker measurements to evaluate temporal changes, in particular in the first days of life, taking into account time points corresponding to routine clinical practice (such as 24 or 48 h from birth).

A key aspect of the study protocol was to ensure the full security of patient information. All data extracted from hospital systems were subjected to a rigorous full anonymisation process before any statistical analyses were performed. All direct identifiers were removed and replaced with unique study-specific codes to ensure data pseudonymisation. This process minimised the risk of patient re-identification, which is in line with the current regulations on the protection of personal data (GDPR) and ethical standards for Big Data research [31,32].

### 2.2. Study Population and Inclusion Criteria

The analysis included cases of newborns with clinically confirmed sepsis, suspected (early) sepsis and healthy patients who underwent serial laboratory testing at predefined time intervals. A prerequisite was the availability of data that allowed plotting the trajectory of changes in the critical diagnostic window 0–24–48 and 72 h from the onset of clinical symptoms or initial clinical evaluation, as applicable. Therefore, cases with missing laboratory data at the predefined time points were excluded from the analysis. Consequently, patients who died before complete serial laboratory data could be obtained were not eligible for inclusion.

### 2.3. Characterisation of the Variables Used

The raw results of laboratory assays were transformed into a set of variables describing both absolute values and their variability over time. For the analysed marker, features covering the entire follow-up period and defined time windows (24 and 48 h from the moment of suspicion of sepsis) were generated. Static variables included basic descriptive statistics, such as minimum values (min), maximum (max) and mean values (mean), calculated both for the entire observation period and in designated time windows. This allowed us to characterise the range and distribution of marker values over time. To capture variability, variables describing the relationships between successive measurements were also determined. These included absolute change in value (abs_change) and relative change (pct_change), which allowed both the scale and direction of the changes to be assessed, considering the individual baseline.

In addition, rate of change indicators (change_rate) was calculated, defined as the change in the value of a parameter per unit of time between successive measurements. These variables constitute a quantitative description of the dynamics of marker changes. The set of variables prepared in this way was used as input data for further statistical analysis and predictive modelling.

### 2.4. Statistical Analysis

Relationships between analysed variables were assessed using correlation analysis. This method was selected due to the lack of assumption about the normality of the distribution of the analysed data and the possibility of assessing monotonic relationships between variables. The strength and direction of the associations between variables were assessed on the basis of the values of the correlation coefficient (r), while statistical significance was determined based on the corresponding *p*-values. The exploratory analysis aimed to identify variables significantly associated with the documented clinical course of neonatal sepsis and to support further interpretation of the findings.

### 2.5. Scope of Data

To provide a complete pathophysiological context for the analysed time series, the anonymised dataset was supplemented with critical clinical, demographic and perinatal variables. Baseline variables such as birth weight, sex and type of delivery were taken into account, together with variables determining the risk of vertical infection, including maternal GBS (group B Streptococcus) status and membrane rupture (PROM/PPROM). These parameters are classic risk factors for early neonatal sepsis (EOS) and their integration with laboratory data may improve the sensitivity of predictive analyses [33,34]. An important element of the dataset was the documentation of pharmacological interventions, including perinatal steroid therapy and antibiotic prevention and therapy implemented both before and at birth [35,36].

A key element of the dataset was multiple time-series laboratory measurements, including inflammatory markers (CRP), haematological parameters (including a leukogram distinguishing between cell subpopulations), biochemical indicators and gasometric parameters. These measurements were collected longitudinally during the first days of the newborn’s life, which made it possible to analyse both absolute values and the dynamics of changes over time. Haematological assessments were derived from routine clinical practice and could include analyses performed with or without differential counts, including manual or automated leukocyte differentiation, depending on clinical and laboratory procedures. The specific analytical methodology was not standardised across all cases and not evaluated separately in the present study.

In addition, dynamic indicators of clinical stability of the neonatal during the early adaptation period were included in the analysis, such as the occurrence of metabolic acidosis in the first three days of life and episodes of tachycardia occurring immediately after birth and during the early neonatal period. Tachycardia and acid–base imbalances are recognised clinical signals of septic shock and multi-organ failure, showing a significant correlation with rapid changes in marker concentrations [37,38]. Taking into account the conditions of care (stay in an incubator) as a categorical variable allowed for the standardising the influence of the external environment on vital parameters [39,40].

## 3. Results

### 3.1. Correlations of Clinical and Perinatal Parameters

In the analysed cohort of newborns (*n* = 122), association between clinical factors and clinical course of sepsis progression rates was statistically verified. In terms of haemodynamic and metabolic stability immediately after birth, significant correlations were shown for postnatal tachycardia (r = 0.46, *p* < 0.001) and the occurrence of metabolic acidosis during the first three days of life (r = 0.45, *p* < 0.001). Organ failure (r = 0.46, *p* < 0.001) and respiratory failure (r = 0.42, *p* < 0.001), as defined in the study protocol, were also significantly associated with the clinical evolution of neonatal sepsis.

### 3.2. C-Reactive Protein Dynamics

**Figure 1:**
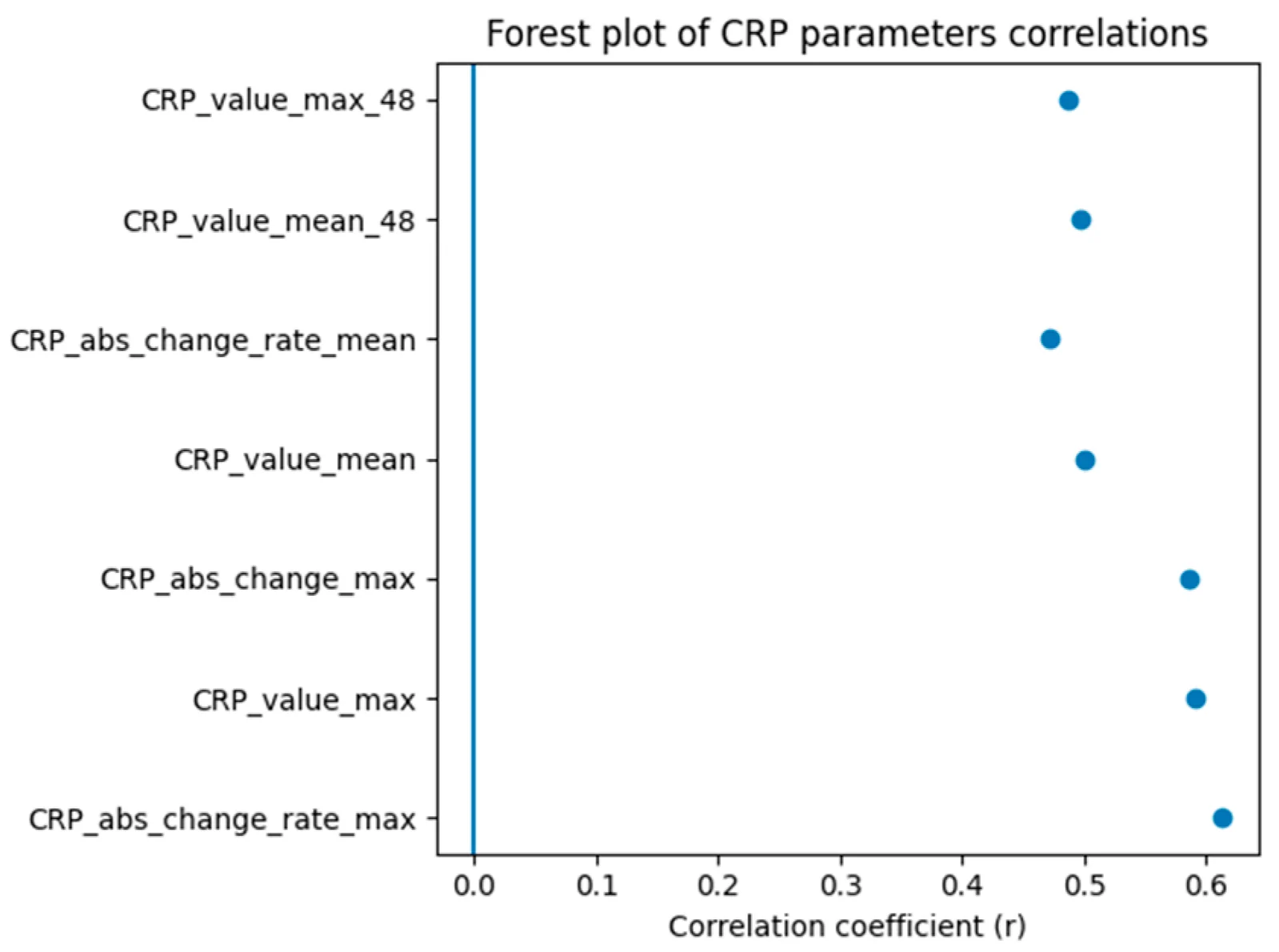
Forest plot of C-reactive protein dynamics associated with the clinical course toward early-onset sepsis (EOS).

Analysis of C-reactive protein (CRP) measurements in newborns allowed for identifying the variables with the highest correlation strength in the entire dataset. The highest correlation coefficient was recorded for the parameter describing the growth dynamics CRP_abs_change_rate_max (r = 0.613, *p* < 0.001). Absolute values reached lower coefficients, respectively for CRP_value_max (r = 0.591, *p* < 0.001) and CRP_abs_change_max (r = 0.587, *p* < 0.001). Time-averaged indicators, such as CRP_value_mean (r = 0.500, *p* < 0.001) and CRP_abs_change_rate_mean (r = 0.472, *p* < 0.001), showed a weaker correlation than the peak-value features. In the analysis taking into account the rigid time window of 48 h, the variables CRP_value_mean_48 (r = 0.498, *p* < 0.001) and CRP_value_max_48 (r = 0.487, *p* < 0.001) retained statistical significance (Figure 1.).

### 3.3. Parameters of the White Blood Cell Parameters

Analysis of leukocyte profiles revealed several significant correlations, with particular emphasis on the fraction of basophils and neutrophils measured during the first 72 h of life. The strongest correlation within peripheral blood count was shown for BASO_%_value_max (r = 0.606, *p* < 0.001). The dynamic parameters of this fraction, including BASO_%abs_change_max (r = 0.553, *p* < 0.001) and the rate of change BASO_%_abs_change_rate_max (r = 0.516, *p* < 0.001), also reached high levels of significance.

**Figure 2:**
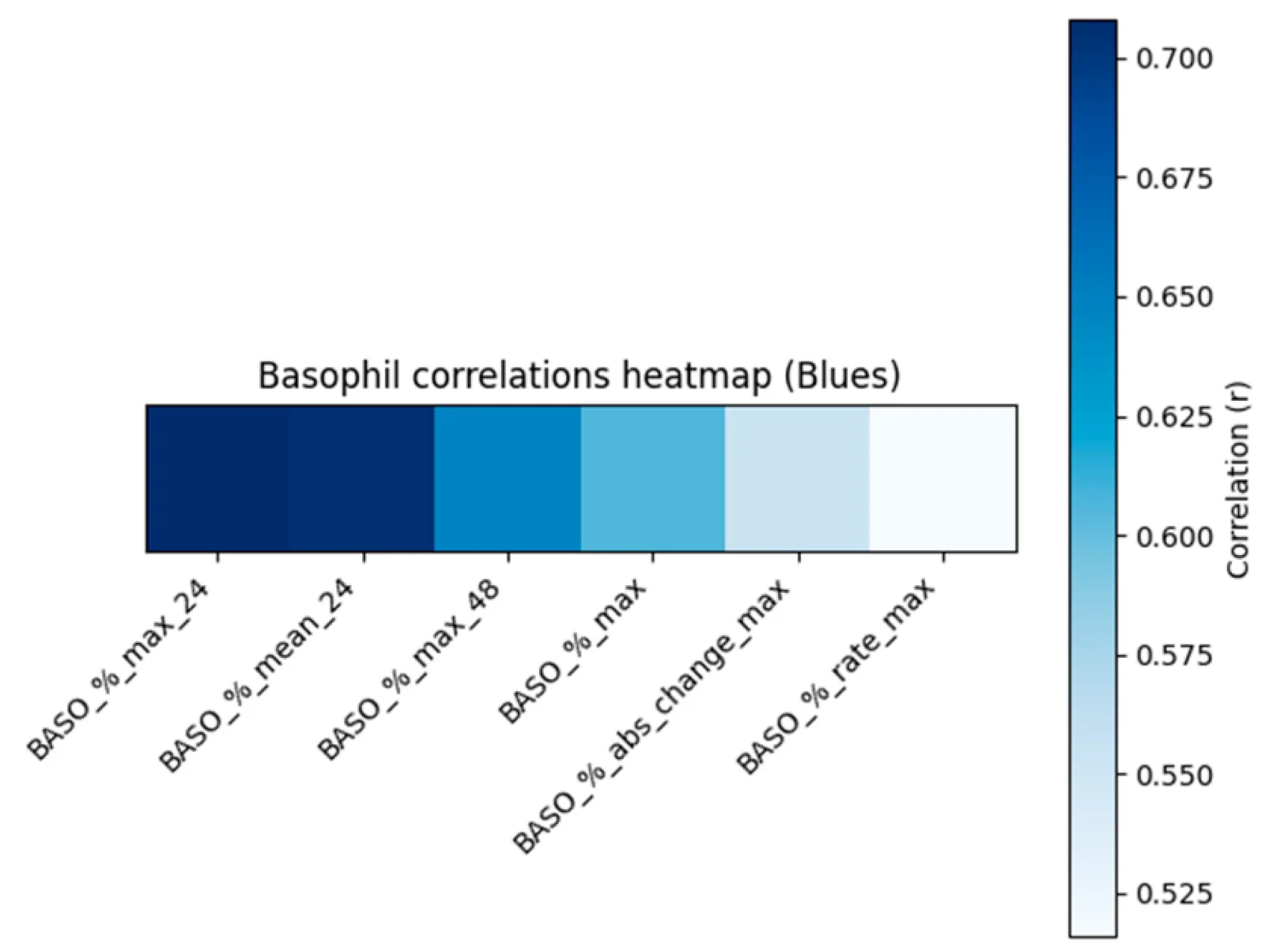
Heatmap of basophil parameters dynamics associated with the clinical course toward early-onset sepsis (EOS).

An extended analysis showed that even stronger relationships were observed within the first 24 h after sepsis was suspected. During this period, basophil values reached the highest correlation coefficients for BASO_%_value_min_24 (r = 0.708, *p* < 0.001), BASO_%_value_mean_24 (r = 0.705, *p* < 0.001) and BASO_%_value_max_24 (r = 0.697, *p* < 0.001) (Figure 2) Within the 48 h window, correlations remained significant but slightly weaker, indicating the particular importance of a very early immune response.

**Figure 3:**
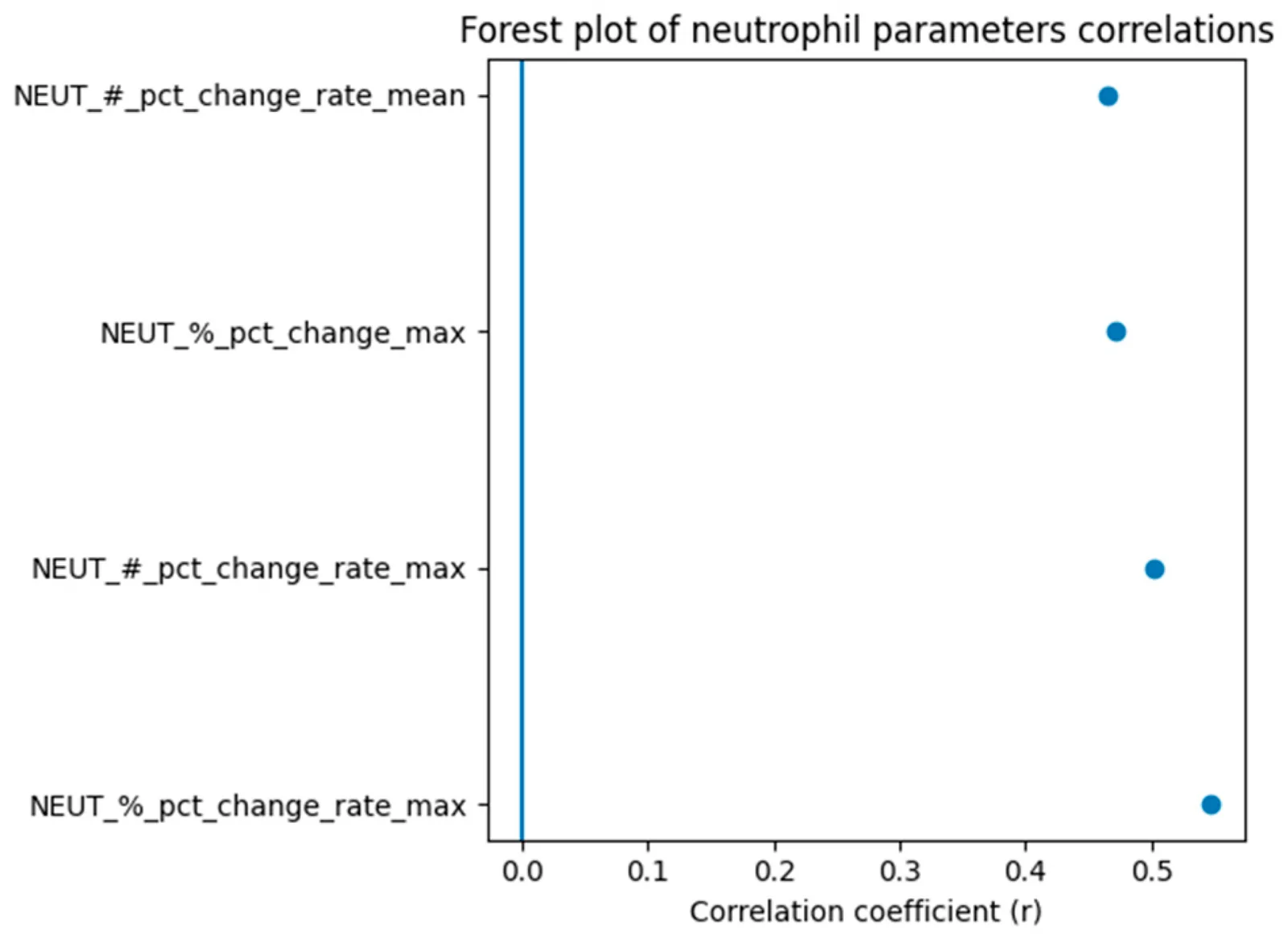
Forest plot of neutrophil parameters dynamics associated with the clinical course toward early-onset sepsis (EOS).

In the neutrophil system, significant associations were noted for NEUT_%_pct_change_rate_max (r = 0.547, *p* < 0.001) and NEUT_#_pct_change_rate_max (r = 0.502, *p* < 0.001). Static parameters such as NEUT_%_pct_change_max showed correlations at (r = 0.472, *p* < 0.001), while the mean rate of change NEUT_#_pct_change_rate_mean reached (r = 0.466, *p* < 0.001). Other white blood cell fractions, including LYMPH_%_abs_change_max lymphocytes (r = 0.493, *p* < 0.001) and monocytes MONO_%_abs_change_max (r = 0.476, *p* < 0.001) showed positive correlations with clinical progression, with a negative correlation for the values of the minimum percentage of monocytes MONO_%_value_min (r = −0.414, *p* < 0.001) (Figure 3).

### 3.4. Analysis of the Red Blood Cell Parameters

**Figure 4:**
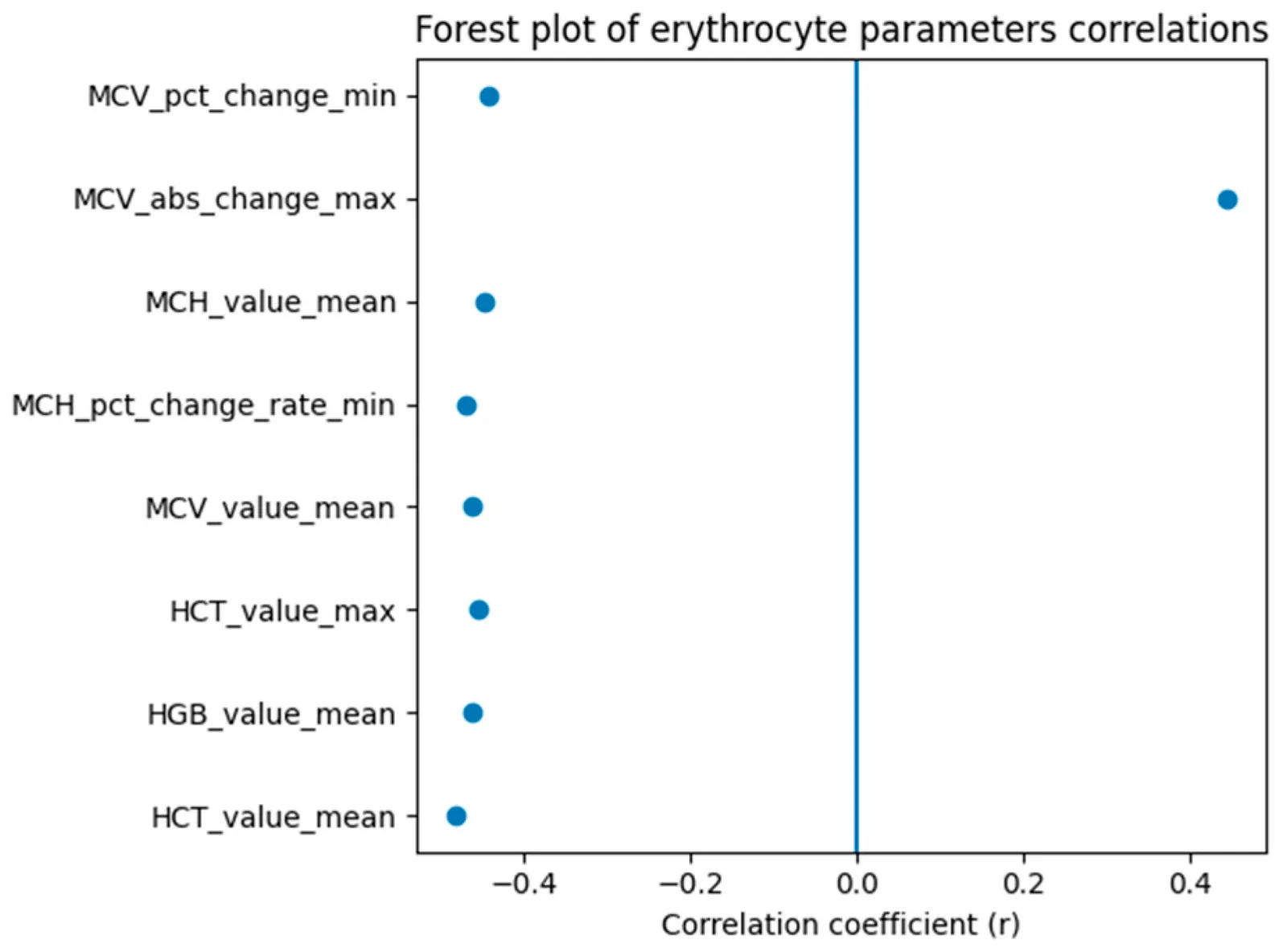
Forest plot of erythrocyte parameters dynamics associated with the clinical course toward early-onset sepsis (EOS).

All erythrocyte-related parameters showed negative correlation coefficients with the analysed endpoints. Significant negative associations were observed for HCT_value_mean (r = −0.482, *p* < 0.001), HGB_value_mean (r = −0.462, *p* < 0.001) and HCT_value_max (r = −0.456, *p* < 0.001). Volume indices and erythrocyte haemoglobin mass also showed a negative correlation for MCV_value_mean (r = −0.464, *p* < 0.001), MCH_pct_change_rate_min (r = −0.469, *p* < 0.001) and MCH_value_mean (r = −0.447, *p* < 0.001). The dynamics of changes in erythrocyte volume was characterised by a positive correlation for MCV_abs_change_max (r = 0.444, *p* < 0.001) and a negative correlation for MCV_pct_change_min (r = −0.442, *p* < 0.001) (Figure 4).

### 3.5. Analysis of the Platelet Parameters

Analysis of platelet parameters revealed a heterogeneous pattern of associations with the analysed endpoints, with the strongest relationships observed for variables reflecting platelet count dynamics. The strongest positive relationships were observed for parameters describing relative changes in platelet counts, including PLT_pct_change_max (r = 0.351, *p* = 0.001) and PLT_pct_change_rate_max (r = 0.329, *p* = 0.002). Statistically significant correlations were also obtained for indicators based on absolute change dynamics, such as PLT_abs_change_rate_max (r = 0.244, *p* = 0.026), PLT_abs_change_rate_mean (r = 0.240, *p* = 0.029) and PLT_abs_change_max (r = 0.227, *p* = 0.039).

**Figure 5:**
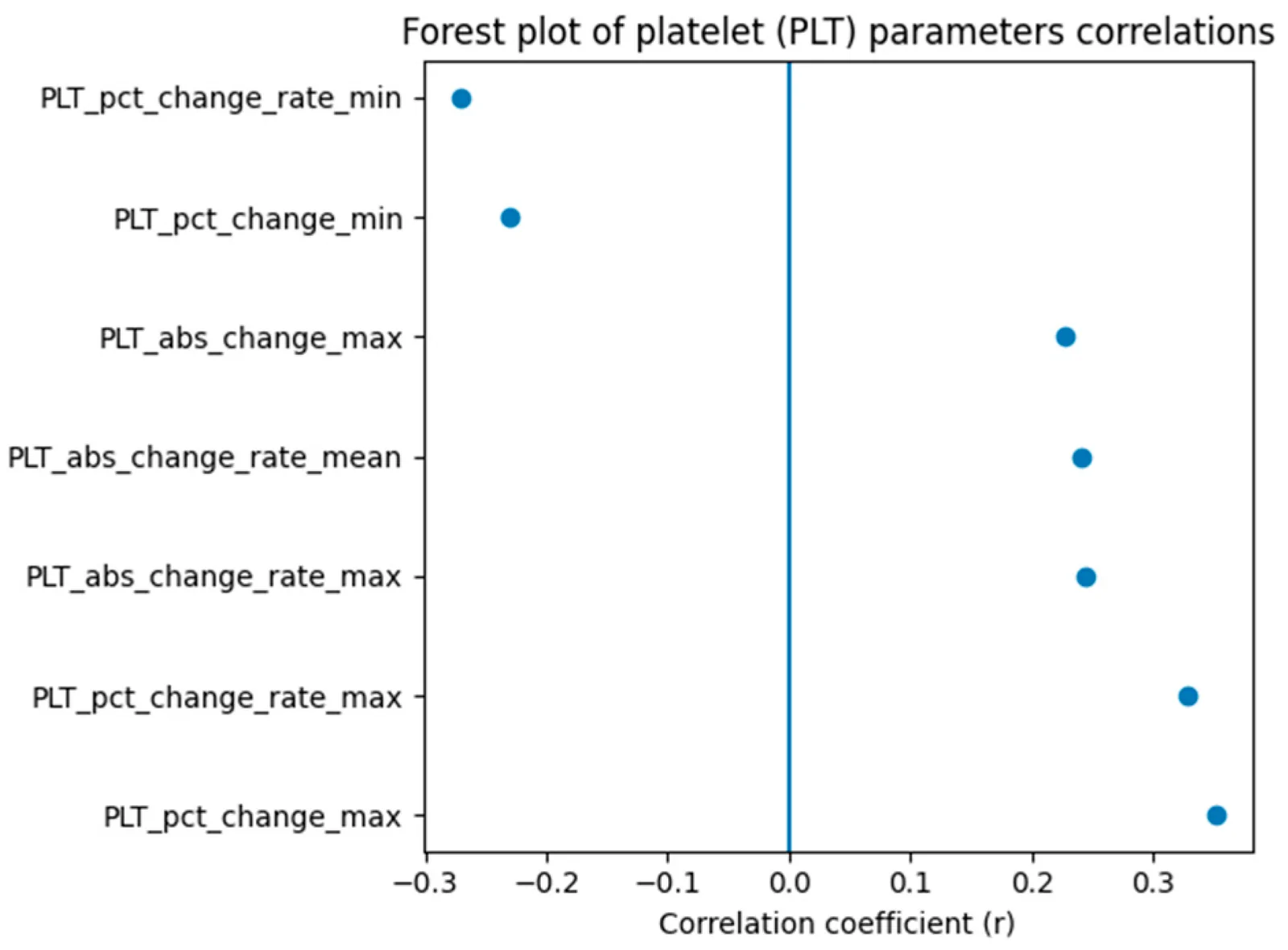
Forest plot of platelet parameters dynamics associated with the clinical course toward early-onset sepsis (EOS).

In contrast, the parameters describing relative decreases showed significant negative correlations, for PLT_pct_change_min (r = −0.231, *p* = 0.036) and PLT_pct_change_rate_min (r = −0.270, *p* = 0.014) (Figure 5).Other variables, including mean values and static platelet count parameters PLT_value_mean, PLT_value_max, PLT_value_min, did not show statistically significant relationships with the analysed endpoints.

### 3.6. Analysis of Blood Gas Parameters

**Figure 6:**
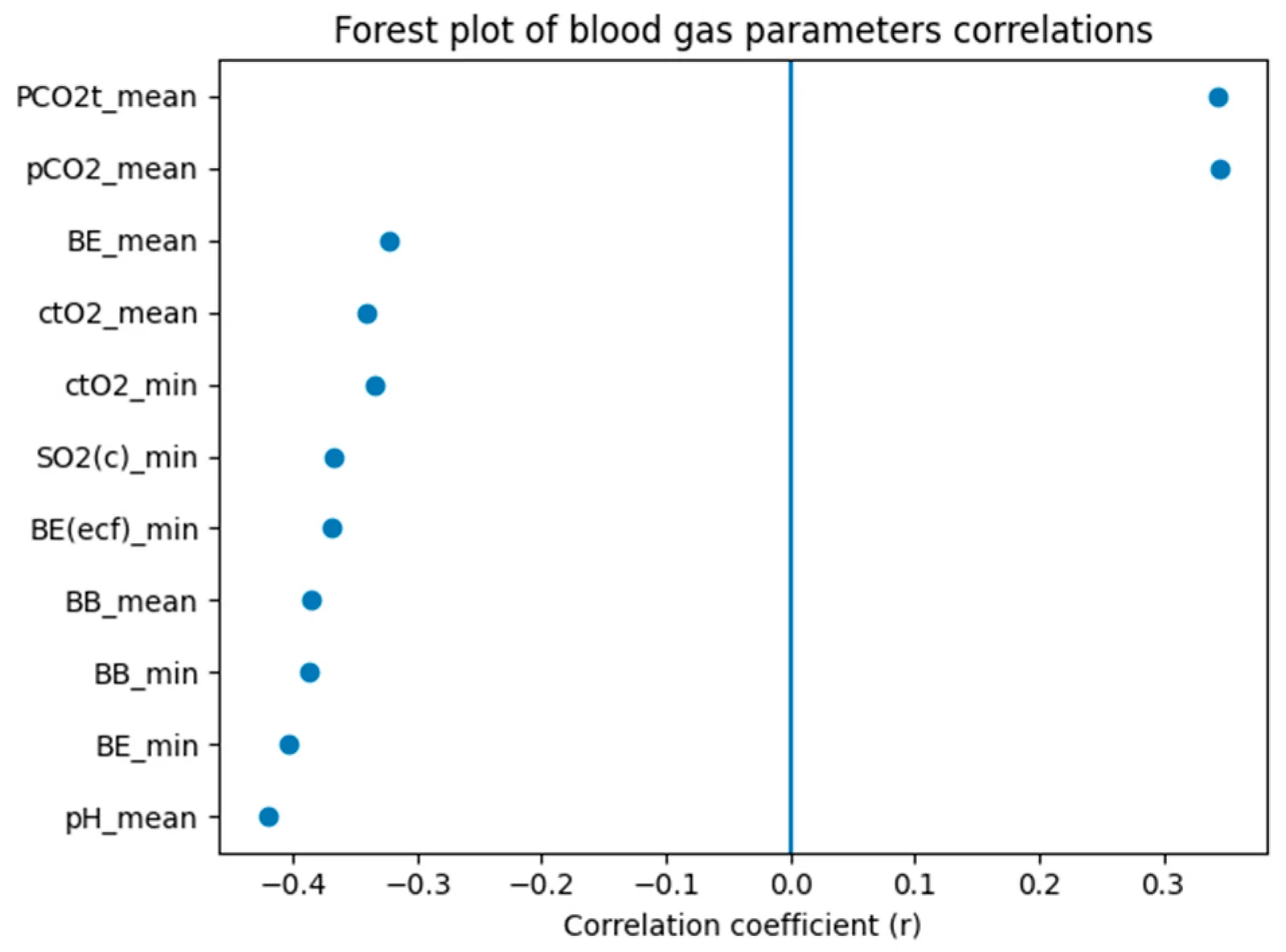
Forest plot of blood gasometry parameters dynamics associated with the clinical course toward early-onset sepsis (EOS).

The analysis of gasometric parameters showed a wide range of important correlations, most of them negative, indicating a relationship between acid–base imbalances and blood oxygenation with the progression of the clinical condition. The strongest relationships were recorded for parameters related to acid–base balance, including pH_value_mean (r = −0.421, *p* < 0.001) and BE_RKZ_value_min (r = −0.403, *p* = 0.001), together with BB_RKZ_value_min (r = −0.387, *p* = 0.002) and BB_RKZ_value_mean (r = −0.385, *p* = 0.002) (Figure 6).

Significant correlations were also observed for blood oxygenation parameters such as SO_2_(c)_value_min (r = −0.367, *p* = 0.003), ctO_2__value_min (r = −0.335, *p* = 0.008) and ctO_2__value_mean (r = −0.341, *p* = 0.007), indicating a relationship between reduced saturation and oxygen content and a more severe clinical course.

The parameters BE (ecf)_value_min (r = −0.369, *p* = 0.003) and BE_value_mean (r = −0.323, *p* = 0.011) also showed significant relationships, emphasising the importance of metabolic disorders in the course of sepsis.

### 3.7. Other Additional Biochemical Parameters

Analysis of biochemical parameters revealed moderate correlations with clinical evolution of sepsis progression, with selected metabolic and electrolyte markers being of the greatest importance. The strongest positive associations were recorded for UREA_value_max (r = 0.383, *p* = 0.001) and GLU_value_max (r = 0.349, *p* = 0.003), while mean blood glucose values showed a weaker but significant correlation (GLU_value_mean, r = 0.267, *p* = 0.026).

**Figure 7:**
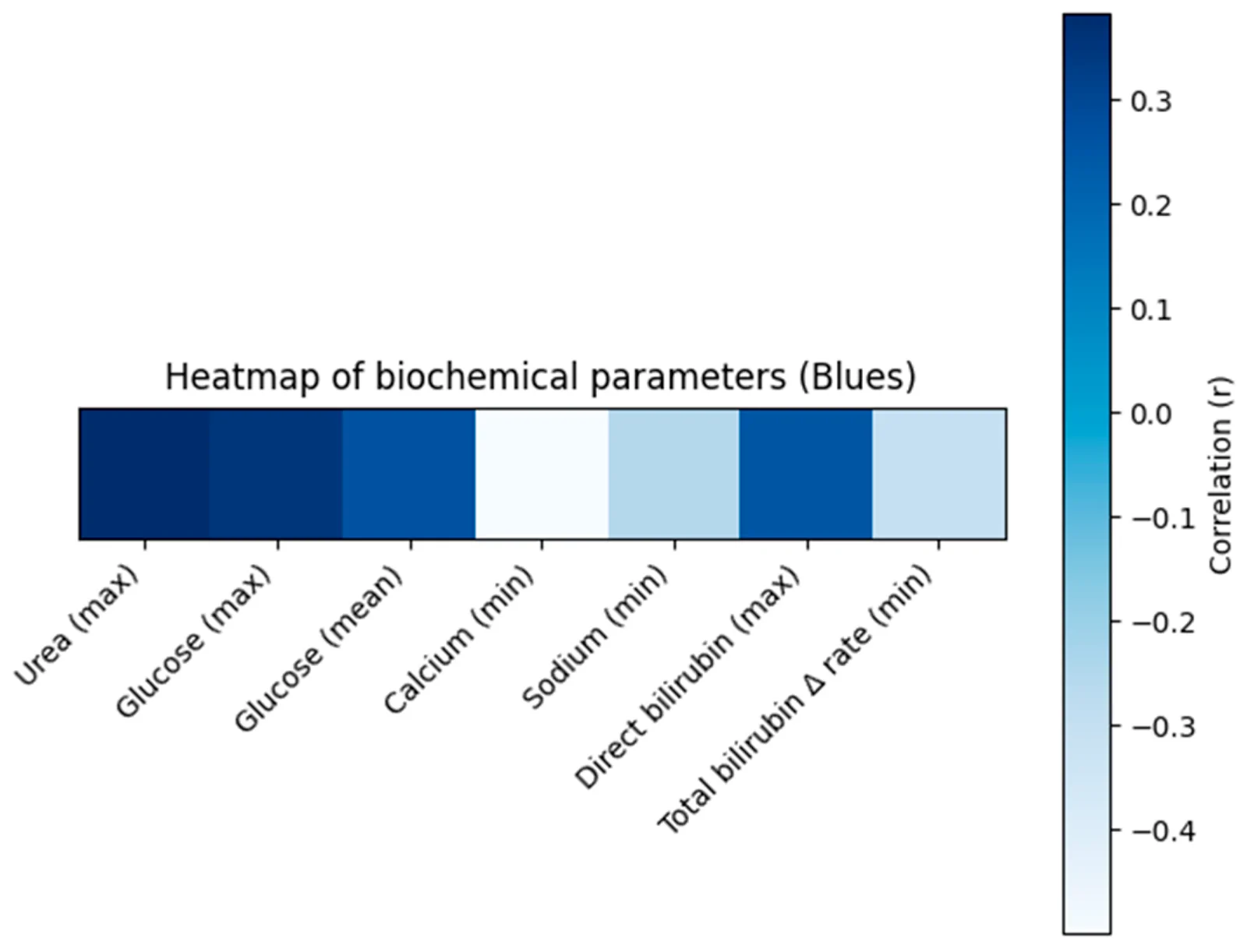
Heatmap of biochemical parameters dynamics associated with the clinical course toward early-onset sepsis (EOS).

Among electrolyte parameters, a negative correlation for Ca_value_min (r = −0.499, *p* < 0.001) was particularly important, indicating a relationship between lower calcium concentrations and the clinical severity. A similar, albeit weaker, direction of association was observed for Na_value_min (r = −0.257, *p* = 0.032). In addition, significant associations have been shown for markers of liver function and bilirubin metabolism, including ALT (GPT)_value_max (r = 0.315, *p* = 0.012) and BIL-D_value_max (r = 0.252, *p* = 0.040). Analysis of the dynamics of total bilirubin changes revealed a significant negative correlation for BIL-T_pct_change_rate_min (r = −0.308, *p* = 0.015) (Figure 7)

Data on statistically insignificant variables (sex, type of birth, body weight) are omitted from this detailed description.

## 4. Discussion

### 4.1. Significance and Impact of the Results Obtained

This study analysed a retrospective cohort of newborns and their mothers (N = 122) evaluated for neonatal sepsis. The analyses included variables with varying levels of data completeness, resulting in parameter-specific sample sizes ranging from 73 to 94 observations. These differences reflected the availability of complete serial measurements for individual laboratory parameters rather than additional patient exclusions, meaning that the same newborn could contribute to some parameter-specific analyses but not to others. No significant associations were found between the birth weight or sex of the newborn (sex variable—female) and the analysed parameters of the course of sepsis.

### 4.2. Study Impact

Analysis of C-reactive protein (CRP) kinetic parameters provided strong evidence supporting the clinical relevance of dynamic biomarker assessment alongside single-point measurements. These findings suggest that the rate of inflammatory response progression may provide additional information on disease dynamics beyond isolated measurements. Importantly, parameters describing the mean dynamics of change, such as CRP_abs_change_rate_mean, demonstrated substantially weaker associations than the values of extreme dynamics. This suggests that it is not so much averaged trends that are crucial, but short-term episodes of rapid acceleration of CRP changes, which may reflect critical moments in the course of the inflammatory response.

In the area of morphological parameters, several variables have been identified that show diagnostic value. Particular attention should be given to basophils, so far rarely exposed in predictive models of sepsis. The BASO_%_value_max variable achieved one of the highest correlation coefficients. At the same time, these findings regarding basophil fractions should be considered exploratory and hypothesis-generating and require independent confirmation using standardised leukocyte differentiation methods.

Equally strong associations were noted in the neutrophil system. However, these parameters and variations in haematocrit should be interpreted cautiously, as they may reflect secondary systemic and iatrogenic alterations associated with critical illness, haemodynamic instability, fluid therapy, or repeated blood sampling rather than a direct predictive association with sepsis onset. Consequently, HCT changes are more likely to represent a downstream manifestation of ongoing inflammatory and haematological dysregulation than an independent early biomarker of neonatal sepsis.

Analysis of platelet parameters showed that the strongest correlations were recorded for relative changes in the number of platelets (PLT_pct_change_max) and their maximum rate (PLT_pct_change_rate_max), while static parameters (PLT_value_mean, PLT_value_max) showed no significant relationships.

Analysis of gasometric parameters revealed a consistent and biologically plausible pattern indicating acid–base imbalances and blood oxygenation. The strongest correlations were noted for parameters related to acidosis, including pH and base excess (BE), which showed significant negative associations with the clinical course. Further, parameters related to CO_2_ retention (pCO_2_) showed positive correlations, reflecting the complementary mechanism of respiratory-metabolic disorders. This pattern of results indicates a consistent picture of homeostatic failure, involving both metabolic and respiratory components. Gasometric measurements were obtained from routine clinical practice and included bedside and laboratory analyses from different sample types, including cord and blood. These procedures were not standardised across the cohort, therefore, gasometric parameters should be interpreted cautiously and primarily within the overall clinical context.

Biochemical parameters showed moderate but consistent correlations with the clinical course, reflecting metabolic and organ disorders associated with sepsis. The strongest positive associations were observed for markers related to metabolism and renal function, such as urea (urea) and glucose, indicating a role for metabolic stress and catabolism. However, elevated urea levels should be interpreted cautiously, as current evidence does not support urea as a specific biomarker of neonatal sepsis. Increased urea concentrations may rather reflect secondary metabolic, renal, haemodynamic, or treatment-related disturbances accompanying critical illness. But decreased calcium levels should be interpreted cautiously, as hypocalcaemia is not considered a specific biomarker of neonatal sepsis and may instead reflect secondary disturbances associated with systemic inflammation, metabolic dysregulation, impaired perfusion, or intensive care interventions in critically ill neonates.

Previous studies have consistently shown that no single inflammatory biomarker provides sufficient diagnostic accuracy for neonatal sepsis. CRP and PCT are the most extensively investigated markers, but their diagnostic performance depends on the timing of measurement and serial assessment is generally more informative than interpretation of an isolated value. Similarly, haematological indices such as neutrophil parameters provide supportive but only moderate diagnostic accuracy. In contrast to studies based mainly on individual measurements or predefined cut-off values, the present study quantified the direction and rate of within-patient laboratory changes.

### 4.3. Limitations

The main limitation of this study is its retrospective nature. The use of anonymised historical data from the Electronic Health Record (EHR) systems of the hospital carries a risk of selection bias and incomplete clinical records. While the database cleaning process and rigorous inclusion criteria have resulted in a homogeneous cohort, any gap in the chronology of laboratory determinations can affect the precision of parameter calculations. This study was based on routinely available biochemical and haematological parameters commonly used in neonatal practice. Consequently, other potentially valuable biomarkers were not included and should be evaluated in future studies. In addition, the analysis did not incorporate longitudinal clinical monitoring data or maternal/perinatal variables, which may further improve early sepsis stratification and predictive modelling. The retrospective nature of the study also makes it impossible to fully control confounding factors such as variability in blood sample collection procedures or differences in the time of sample transport to the laboratory. Given the exploratory and hypothesis-generating nature of the present analysis, no adjustment for multiple comparisons was applied. This approach was consistent with the primary objective of broadly identifying clinically relevant candidate features and temporal patterns for subsequent confirmatory studies, rather than establishing definitive predictive relationships at this stage.

In addition, the analysis was deliberately based on biochemical and morphological parameters available in routine practice, but it should be acknowledged that this does not account for other potentially important markers (e.g., presepsin or modern CD64 indicators), which could describe the clinical evolution of sepsis progression vector even more precisely. This should be analysed in subsequent projects.

## 5. Conclusions

### 5.1. New Perspectives in Neonatal Sepsis Diagnostics

The principal finding of this study is confirmation of the hypothesis that dynamically analysed parameters have superior diagnostic value to traditional point measurements. The correlation between the maximum rate of changes in inflammatory markers and the severity of the clinical course proves that neonatal sepsis should not be diagnosed solely based on exceeding static cut-off thresholds, but primarily by analysing the vector of their increase [40]. The rate of increase in the concentration of markers reflects the dynamics of inflammatory cascade activation and the intensity of the body’s immune response, which allows for the identification of the critical phase of infection much earlier than is the case when waiting for peaks to be reached [41]. The introduction of the concept of a critical variable based on the concentration derivative over time is a key step towards personalising the decision-making process in neonatal intensive care units.

### 5.2. Dynamics of Parameter and Significance

This study showed that clinical evolution of sepsis progression is characterised by complex multidimensional alterations in the biochemical and morphological profile that goes beyond isolated fluctuations in individual parameters [42]. The predictive value of biomarkers depends not only on their absolute levels, but primarily on their temporal trajectories and rates of change. From a practical perspective and the development of analytical methods, these results highlight the need to move away from static diagnostic models towards a time-series analysis-based approach. Considering the dynamics of parameter changes is a key element in the designing modern predictive systems, including artificial intelligence-based solutions that operate on change trajectories and relationships between variables. In addition, our findings should be interpreted within the context of modern neonatal care, where early sepsis recognition relies not only on laboratory biomarkers but also on longitudinal clinical assessment and non-invasive monitoring. Temporal analysis of routinely available inflammatory markers may support clinical decision-making while remaining compatible with standard neonatal practice.

Additionally, the clinical relevance of early dynamic assessment may extend beyond the immediate diagnosis of EOS. Neonatal sepsis is associated with subsequent morbidity and adverse neurodevelopmental outcomes and recent evidence indicates that these consequences may occur after both early- and late-onset infection. Thus, early characterisation of the inflammatory and physiological response may contribute not only to acute risk assessment but also to identifying infants who require closer clinical surveillance. However, the generalisability of the present findings may be limited by differences in gestational and postnatal age, birth weight, pathogen spectrum, maternal and perinatal exposures, laboratory methods, sampling intervals and local NICU practices. Prospective validation in independent populations, including infants with LOS and extremely preterm neonates, is therefore necessary before these dynamic variables can be incorporated into clinical decision-support models.

### 5.3. Foundations for Predictive Medicine and Artificial Intelligence

The developed and verified critical variables provide a conceptual and mathematical apparatus for a new generation of clinical decision-support systems. Demonstrating the high significance of parameters while reducing information noise defines the optimal input data architecture for advanced machine learning algorithms [43]. The results of the study clearly indicate that the future of neonatal sepsis diagnostics lies in the integration of heterogeneous retrospective and real-time data, where artificial intelligence will be able to process the defined in this work [44]. This analysis represents an exploratory step toward identifying candidate variables for future predictive modelling, which will be the subject of subsequent stages of research work.

## Data Availability

The original contributions presented in this study are included in the article. Further inquiries can be directed to the corresponding author.
